# Plasma levels of tumor necrosis factor-α in adolescent idiopathic scoliosis patients serve as a predictor for the incidence of early postoperative cognitive dysfunction following orthopedic surgery

**DOI:** 10.3892/etm.2015.2241

**Published:** 2015-01-30

**Authors:** XU ZHENG, ZHENGLIANG MA, XIAOPING GU

**Affiliations:** Department of Anesthesiology, Drum Tower Hospital, Medical Department of Nanjing University, Nanjing, Jiangsu 210008, P.R. China

**Keywords:** postoperative cognitive dysfunction, biomarker, plasma, tumor necrosis factor-α, cortisol

## Abstract

The aim of the present study was to evaluate whether the levels of the plasma biomarkers, cortisol, interleukin (IL)-1β, IL-6, IL-10 and tumor necrosis factor (TNF)-α, change in adolescent idiopathic scoliosis patients with postoperative cognitive dysfunction (POCD); thus, may serve as predictive factors for POCD. In total, 75 adolescent scoliosis patients, aged between 11 and 18 years and categorized as American Society of Anesthesiologists classification I or II, were scheduled for orthopedic surgery with general anesthesia. Blood samples were collected on the day of admission and at day 2 following surgery. The plasma levels of IL-1β, IL-6, IL-10 and TNF-α were measured using an enzyme-linked immunosorbent assay, while the concentrations of cortisol were determined using a radioimmunoassay. Cognitive function was assessed one day prior to and at day 7 following the surgery in a quiet room with the guidance of a Chinese version protocol. In total, 66 patients completed the sample collection and neurocognitive tests. According to the criteria for the diagnosis of POCD, 19 patients (28.8%) developed POCD within seven days of surgery. No statistically significant differences were observed in the baseline concentrations of cortisol, TNF-α, IL-1β, IL-10 or IL-6 between the POCD and non-POCD groups. However, the baseline plasma level of TNF-α on day 2 in the POCD group was found to be higher compared with the non-POCD group. In addition, at day 2 after surgery, the concentration of cortisol in the non-POCD group was higher compared with the POCD group. Therefore, the plasma level of TNF-α in adolescent idiopathic scoliosis patients at day 2 following orthopedic surgery may be a predictor for the incidence of early POCD.

## Introduction

Postoperative cognitive dysfunction (POCD) is a common complication following major surgery, particularly in elderly individuals. POCD is defined as a decline in a variety of neuropsychological functions, including memory, executive functioning and speed of processing, and is often accompanied by a decrease in social skills. POCD may last for several months or years and affect the quality of life, or may even manifest as a permanent cognitive decline marked by further deterioration (1). Although the etiology and pathophysiology of POCD are not fully understood, several risk factors for POCD have been identified, including the type of surgery, the extent of surgical trauma and the stress response, while increased age has been consistently reported as a risk factor (2–4).

Adolescent idiopathic scoliosis affects 1–3% of the population at risk (children aged between 10 and 16 years), and is a common disease in adolescent orthopedic hospital departments, usually requiring surgical treatment (5). Idiopathic scoliosis surgical treatment is often accompanied by a large trauma, a large amount of bleeding and extensively prolonged surgery duration. In addition, a wake-up test is often required during surgery for early detection of spinal cord injuries. These factors induce intensive stress responses to the patients. In addition, Welsh *et al* found that verbal fluency, action sequences and complex planning skills may not mature in adolescents until the age of 12 or later (6). Therefore, it may be hypothesized that these adolescents have an elevated risk for POCD, following orthopedic surgery.

In the present study, variations in the levels of plasma markers, including cortisol, interleukin (IL)-1β, IL-6, IL-10, and tumor necrosis factor-α (TNF-α), were investigated to determine whether these markers are potential predictive factors for the development of POCD in patients with adolescent idiopathic scoliosis following surgery.

## Materials and methods

### Subjects

The study was performed between July 2012 and March 2013 on 75 adolescent scoliosis patients aged between 11 and 18 years. The patients had been categorized as American Society of Anesthesiologists (ASA) classification I or II (7), and were scheduled for orthopedic surgery with general anesthesia induced by total intravenous anesthesia. The patients did not suffer from severe hypotension, hypoxemia or other serious incidents, such as cardiac arrest. Patients with severe congenital, mental and neurological diseases, organ dysfunction, including the liver and kidney, and hearing and visual impairments, as well as those unwilling to comply with the protocol or procedures, unable to understand Mandarin Chinese and with a mini-mental state examination score of <23, were excluded from the study. Among the 75 adolescent scoliosis patients participating in the study, two patients obtained a score of <23 in the mini-mental state examination, one patient had severe hearing impairment, four patients did not complete the cognitive function test within seven days of surgery and two patients did not complete the sample collection. Therefore, 66 patients completed the sample collection and neurocognitive tests. All the procedures were approved by the Ethics Committee of the Drum Tower Hospital of Nanjing University (Nanjing, China). Informed consent was obtained from all the patients or the patients’ families.

### Anesthesia and postoperative analgesia

All the patients followed the routine ‘nothing by mouth’ after midnight or 6 h prior to surgery. The patients were not administered any sedatives or other medicine. The patient’s electrocardiogram, pulse oximetry and invasive blood pressure were continuously monitored during anesthesia. Radial artery catheterization for perioperative blood pressure monitoring and internal jugular vein catheterization for central venous pressure monitoring were required. Induction of anesthesia was achieved by midazolam (0.1 mgkg^−1^), propofol (1.5 mgkg^−1^), vecuronium (0.15 mgkg^−1^) and fentanyl (6 μgkg^−1^). Maintenance of anesthesia was achieved by propofol (4–12 mgkg^−1^h^−1^), dexmedetomidine (0.2 μgkg^−1^h^−1^), atracurium (5 μgkg^−1^min^−1^) and remifentanil (0.2 μgkg^−1^min^−1^). All anesthetics were withheld during the wake-up test, which was performed smoothly in all cases with the patients cooperating well. Following the wake-up test, propofol (4–6 mgkg^−1^h^−1^), dexmedetomidine (0.2 μgkg^−1^h^−1^), atracurium (5 μgkg^−1^min^−1^) and fentanil (2 μgkg^−1^) were used to maintain anesthesia. For patients with bradycardia, administration of 0.05–0.1 mg atropine was required.

Fentanil (3 μgkg^−1^) was routinely administered to each patient 15 min prior to the end of the surgery. All patients received the same postoperative pain control protocol, namely, patient-controlled analgesia (a constant infusion rate of 2 ml/h with a lock time of 15 min), with administration of fentanil (12.5 μgkg^−1^) and ondansetron (8 mg) for two days.

### Enzyme-linked immunosorbent assay (ELISA) and radioimmunoassay

Blood samples were collected at 6:00 AM on the day of admission and at day 2 following surgery. After centrifugation at 470 × g for 10 min, the plasma samples were collected and stored at −70°C for future use. The plasma levels of IL-1β, IL-6, IL-10 and TNF-α were measured using ELISA kits (Yunhan Biotechnology Co., Ltd., Shanghai, China), according to the manufacturer’s instructions. Cortisol concentrations were determined using a radioimmunoassay (Department of Nuclear Medicine, Drum Tower Hospital).

### Preoperative evaluation

The patient age, gender, height, body weight, education and other pertinent information were recorded. The subjective pain of the patients was assessed with a 10-point linear visual analog scale, where 0 represented ‘no pain’ and 10 represented ‘severe pain’. The patients received training in the use of the visual analog scale. Pain scores were determined on day 1 and day 7 after the surgery (8).

### Cognitive function measurement

Cognitive function was assessed one day prior to and at day 7 following surgery in a quiet room, using a cognitive function test in Chinese (9,10). This test battery was designed according to the International Study for Postoperative Cognitive Dysfunction (11), as well as the situation of the patients. A mini-mental state examination was performed first as a screening approach, assessing the orientation, memory and ability to follow instructions. Patients obtaining a score of <23 were excluded from the study (12,13). Next, a visual and verbal learning test was performed to evaluate the word learning and memory abilities of the patients (14). The patients were required to memorize 10 words by studying the words three times, and recall as many words as possible after 20 min. The number of words recalled was recorded. During the 20-min period, a two-part Stroop color-word test was performed to evaluate the executive function of the patients (15,16). In the first part, the patients were required to read aloud 30 color names, while in the second part, the patients were required to name 30 colored patches. The time taken to complete each part of the test and the number of errors made were recorded.

A digit span test was used to assess the short-term memory of the patients (17). In addition, a trail making test required the patients to cross out digits in ascending order (range, 1–25), which were randomly distributed in circles (18). The time taken to complete the task was measured and the number of errors made was counted (17,18). Finally, a number-symbol test was performed, in which the patients were asked to match number and symbol pairs as quickly as possible within the required time of 90 sec (19). The amount of correctly matched pairs were recorded for each patient.

POCD was characterized as the deterioration of one standard deviation (SD) compared with the preoperative test results, obtained from at least two of the aforementioned tests (referred to as ‘the 1-SD criterion’) (2,20). Thereafter, the patients were divided into the POCD and non-POCD groups depending on whether POCD occurred within seven days of the surgery.

### Statistical analysis

Statistical analysis was performed using SPSS 16.0 software (SPSS, Inc., Chicago, IL, USA). The data are presented as the mean ± SD. Intergroup comparisons were conducted by independent-sample t-tests, while intragroup comparisons were analyzed by paired-sample t-tests. Categorical variables were analyzed using χ^2^ or Fisher’s exact tests. In addition, binary logistic analysis was used to investigate the risk factors for the development of POCD. Intergroup comparisons of measurement data with non-normal distribution were performed using the Mann-Whitney U test. P<0.05 was considered to indicate a statistically significant difference.

## Results

### Demographic data

Demographic data of the patients are shown in Table I. No statistically significant differences were observed between the POCD and non-POCD groups with regard to the age, gender, height, body weight, years of education, basic mini-mental state examination scores, ASA classification and length of surgery. In addition, no statistically significant differences were identified in the visual analog scale scores of the two groups at day 1 and day 7 after surgery (P>0.05). These results demonstrated that there was no statistically significant difference in the demographic data of the two groups.

### Identification of patients with POCD according to the International Study for Postoperative Cognitive Dysfunction

To identify patients with POCD, cognitive function measurements were performed with a test battery designed according to the International Study for Postoperative Cognitive Dysfunction and the actual situation of the patients. According to the POCD criteria, 19 patients (28.8%) were diagnosed with POCD within seven days of the surgery (Table II). The patients in the POCD and non-POCD groups were subsequently enrolled in further tests.

### TNF-α may be a risk factor for the indication of POCD

**Figure 1:**
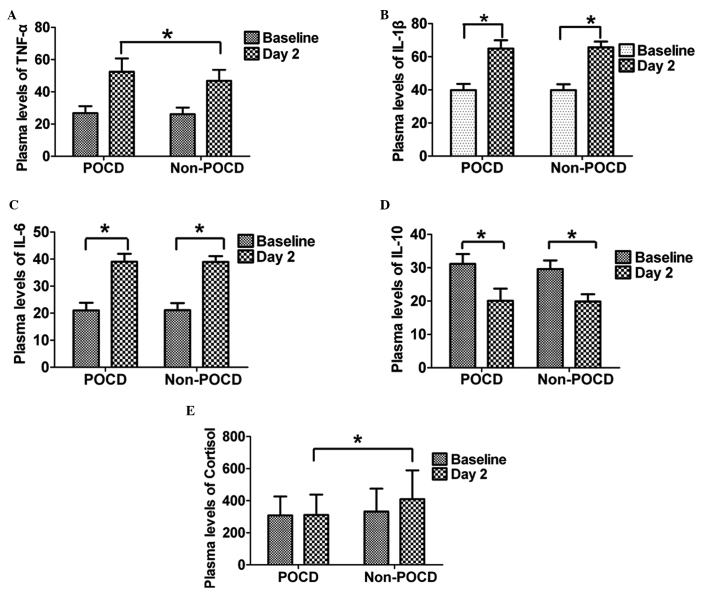
Plasma levels of (A) TNF-α, (B) IL-1β, (C) IL-6 and (D) IL-10 in POCD and non-POCD patients were analyzed using an enzyme-linked immunosorbent assay at baseline and day 2 after surgery. (E) Cortisol concentrations were determined using a radioimmunoassay. Data are presented as the mean ± standard deviation. ^*^P<0.05. POCD, postoperative cognitive dysfunction; TNF, tumor necrosis factor; IL, interleukin.

An ELISA was used to measure the plasma levels of inflammatory mediators. At day 2 after surgery, the plasma levels of IL-1β, IL-6 and TNF-α in the POCD and non-POCD groups were higher when compared with the baseline levels (P<0.05), and no statistically significant differences were observed in the baseline levels between the two groups. At day 2 following surgery, the plasma level of TNF-α in the POCD group was higher compared with the non-POCD group (P<0.05; Fig. 1A). However, no statistically significant differences were observed in the plasma levels of IL-1β and IL-6 between the POCD and non-POCD groups (P>0.05; Fig. 1B and C). By contrast, the plasma levels of IL-10 for patients in the POCD and non-POCD groups at day 2 after surgery were lower than the baseline levels; however, no statistically significant difference was observed (P>0.05; Fig. 1D). The plasma level of TNF-α changed significantly and binary logistic analysis demonstrated that the elevation of TNF-α at day 2 after surgery may be a risk factor for the occurrence of POCD (P=0.018; odds ratio, 1.108; 95% confidence interval, 1.017–1.207).

### Cortisol concentration may not be a suitable indicator for the occurrence of POCD

A radioimmunoassay was employed to determine the concentration of cortisol. No statistically significant difference was identified in the baseline concentrations of cortisol between the POCD and non-POCD groups. However, at day 2 following surgery, the concentration of cortisol in the non-POCD group was found to be higher compared with the POCD group (P<0.05; Fig. 1E). Using the Mann-Whitney U test, the difference in the changing cortisol concentrations between the two groups was investigated (changing level = preoperative cortisol concentration - postoperative concentration). The difference in the pre- and postoperative cortisol concentrations was found to be more significant in the non-POCD group compared with the POCD group. Taking these factors into account in the binary logistic analysis, the reduced cortisol concentration in POCD patients indicated that cortisol may not be a suitable indicator for the occurrence of POCD.

## Discussion

In the present study, a degree of inflammation was found in all the patients following idiopathic scoliosis surgery. At day 2 after surgery, the plasma level of TNF-α in the POCD patients was found to be significantly higher when compared with the non-POCD patients. In addition, non-POCD patients exhibited significantly higher postoperative concentrations of cortisol when compared with the POCD patients.

In previous studies, the incidence of POCD at day 7 after surgery varied widely (11,21). The conflicting results may be attributed to the different criteria for the diagnosis of POCD, as well as the different study populations and types of surgery (22). To the best of our knowledge, no studies exist on POCD in adolescents.

POCD is increasingly recognized as a common complication following major surgery; however, the exact pathophysiology remains unclear. Previous studies have primarily focused on the risk factors associated with early POCD (23–25). In terms of baseline factors associated with the demographic data of the patients, an increased age and lower level of education have been identified as the main risk factors for POCD, according to the International Study for Postoperative Cognitive Dysfunction (1,11). However, the patients enrolled in the present study were not of increased age or lower education levels, and a number of patients developed POCD, which may be due to a variety of reasons. Adolescent scoliosis orthopedic surgery often leads to a large trauma and a large amount of bleeding during the surgery. Cibelli *et al* found that surgery may result in complex systemic responses, including neuroinflammation (26). Systemic and neural inflammation, as a result of surgery, may directly affect the cognitive outcomes of patients (1,27,28). To a certain extent, this observation concurs with the results of present study, which demonstrated that the plasma level of TNF-α in POCD patients was significantly higher compared with non-POCD patients at day 2 following surgery. However, the present study only measured the extent of peripheral inflammation. Terrando *et al* demonstrated in preclinical experiments that peripheral surgery disrupts the blood-brain barrier through the release of TNF-α, which facilitates the migration of macrophages into the hippocampus and impairs cognitive function (29). The results of the present study revealed that inflammatory responses were more severe in POCD patients compared with non-POCD patients. In addition, blood loss and tissue injury in orthopedic procedures may stimulate the immune system to produce more cytokines, increasing inflammatory responses. Data from preclinical studies have supported the hypothesis that inflammation is a possible pathogenic mechanism for POCD (30–32).

The stress response is a physiological reaction for survival, occurring in response to perceived harmful events, attacks or threats (33), and is recognized as the first stage of a general adaptation syndrome. Moderate stress is beneficial to the body, helping to maintain homeostasis and increasing the ability to adapt to the environment. However, an extensive or intense stress response is potentially harmful (34). A large number of studies have hypothesized that excessive stress may be a possible pathogenic mechanism for the development of POCD (35). Bisschop *et al* indicated that high levels of cortisol were closely associated with cognitive decline (4). In addition, Ji *et al* revealed that the plasma cortisol concentration of elderly POCD patients was higher compared with elderly non-POCD patients at day 7 after hip fracture surgery, performed with spinal anesthesia (36). In the present study, the baseline cortisol concentrations were found to be similar in POCD and non-POCD patients. However, at day 2 following surgery, the level of cortisol in the non-POCD group was higher compared with the POCD group. The self-regulation ability of the immune system in POCD patients was hypothesized to be abnormal; thus, adaptive changes were unable to be made to avoid the risks of intense stress. As the pathophysiology of adolescent idiopathic scoliosis is unknown, the present study indicates that the adolescent population may be susceptible to POCD.

In conclusion, the observations of the present study indicated that high plasma levels of TNF-α in patients with adolescent idiopathic scoliosis at day 2 after surgery can predict the incidence of early POCD. However, a large number of clinical studies are required to clarify the role of TNF-α as a predictive factor for POCD.

## Figures and Tables

**Table I:** Demographic data of the POCD and non-POCD patients.

Admission characteristics	POCD (n=19)	Non-POCD (n=47)
Age (years)	14±2	14±2
Height (cm)	158±4	160±7
Body weight (kg)	46±9	48±9
Gender, M/F (n)	6/13	13/34
Education (years)	7.8±1.8	7.6±1.6
ASA classification, I/II (n)	3/16	7/40
Length of surgery (min)	264±60	270±60
MMSE scores	29.2±1.1	29.2±1.0

Values are presented as the mean ± standard deviation. There were no statistically significant differences in the demographic data between the two groups. POCD, postoperative cognitive dysfunction; M, male; F, female; ASA, American Society of Anesthesiologists; MMSE, mini-mental state examination.

**Table II:** Number of patients with >1-SD decline in the test battery at day 7 following surgery.

>1-SD decline	Patients (n=66)
Two tests	13
Three tests	5
Four tests	1
Five tests	0

SD, standard deviation.
